# Care coordination for severe mental health disorders: an analysis of healthcare provider patient-sharing networks and their association with quality of care in a French region

**DOI:** 10.1186/s12913-020-05173-x

**Published:** 2020-06-17

**Authors:** Coralie Gandré, Laurent Beauguitte, Alexandre Lolivier, Magali Coldefy

**Affiliations:** 1 Institut de recherche et documentation en économie de la santé (IRDES), 117 bis rue Manin, 75019 Paris, France; 2 UMR Géographie-cités, Centre National de la Recherche Scientifique, Paris, France

**Keywords:** Mental health care, Coordination, Provider patient-sharing networks, Quality of care

## Abstract

**Background:**

For patients with multiple and complex health needs, such as those suffering from mental health disorders, outcomes are determined by the combined actions of the care providers they visit and their interactions. Care coordination is therefore essential. However, little is known on links between hospitals providing psychiatric care and community-based care providers which could serve as a basis for the creation of formal mental care networks supported by recent policies. In this context, we first aimed to identify and characterize existing types of healthcare provider patient-sharing networks for severe mental health disorders in one French region. Second, we aimed to analyse the association between their characteristics and the quality of the care they provide.

**Methods:**

Patient flows among healthcare providers involved in treating severe mental health disorders in the *Provence-Alpes-Côte-d’Azur* region were extracted from the French national health data system, which contains all billing records from the social health insurance. Healthcare provider networks that have developed around public and private non-profit hospitals were identified based on shared patients with other providers (hospitals, community-based psychiatrists, general practitioners and nurses). Hierarchical clustering was conducted to create a typology of the networks. Indicators of quality of care, encompassing multiple complementary dimensions, were calculated across these networks and linked to their characteristics using multivariable methods.

**Results:**

Three main types of existing healthcare provider networks were identified. They were either networks strongly organized around the main hospital providing psychiatric care; scattered networks involving numerous and diverse healthcare providers; or medically-oriented networks involving mainly physician providers. Few significant associations between the structure and composition of healthcare provider networks and indicators of quality of care were found.

**Conclusions:**

Our findings provide a basis to develop explicit structuring of mental care based on pre-existing working relationships but suggest that healthcare providers’ patient-sharing patterns were not the main driver of optimal care provision in the context explored. The shift towards a stronger integration of health and social care in the mental health field might impact these results but is currently not observable in the administrative data available for research purpose which should evolve to include social care.

## Background

For patients with multiple and complex health needs, in particular those suffering from chronic disorders, outcomes are influenced by the joint actions of all care providers they visit, as well as their interactions [1]. Care coordination, defined as the deliberate organization of patient care activities between different professionals to facilitate appropriate care delivery and meet patients’ needs [2, 3], is therefore essential for a well-functioning healthcare system.

Improving the coordination of care providers, by reinforcing formal care pathways or introducing financial incentives, has been at the heart of healthcare reforms worldwide [4–7]. It is particularly essential for people with mental health disorders as these are complex chronic conditions with long-term adverse consequences which often involve a large number of providers (specialized care, primary care, social services …) [8].

In France, public and private non-profit hospitals with an activity in psychiatry have historically been the main entry point to specialized care for severe mental health disorders. Initially they were all specialized in psychiatry and the integration of psychiatric services in general hospitals only began in the 1980’s [9]. These hospitals are in charge of providing care to cover the mental health needs of the population of given catchment areas with administratively defined limits (psychiatric sectors) [10, 11]. While such care does not only encompass inpatient care, all specialized outpatient care activities available in the public system are developed by multidisciplinary teams managed by hospitals. Therefore, the territorial organization of French mental healthcare has been centered around hospitals, more so than in many other developed countries [9]. This has not encouraged a culture of coordination with other ambulatory care providers. In order to reduce the fragmented nature of the care delivery system, all mental care providers have recently been required to create formal territorial networks (“*projets territoriaux de santé mentale*”, PTSM) in charge of prevention, diagnosis, care, rehabilitation and social integration within regions [12]. They expand beyond psychiatric sectors whose missions were redefined in 2016 to focus on the provision of local ambulatory care, on geographical and financial accessibility to care and on care continuity and coordination [13]. The PTSM reform specifically supports task transfer to other providers including private and non-specialized healthcare (such as primary care providers) and social care to guarantee cross-sectional continuity of care. This reform is very similar to other reforms recently implemented to promote the development of formal networks for the care of mental health disorders in several Western countries with the aim of supplying comprehensive care to all mental health users. This is in particular the case in Canada and Belgium, where previous research using survey data has demonstrated that network structure was associated both with the effective implementation of networks and patients’ outcomes [14–16]. This research has also underscored the potential impact of different organizational contexts on the robustness of findings as well as the complexity of quantifying coordination between healthcare actors on a large scale [14]. In France, there is little empirical knowledge regarding the links between hospitals providing psychiatric care and community-based health professionals which could serve as a basis for the development of optimal formal networks. Identifying naturally occurring healthcare provider networks based on the patients they share – prior to the implementation of formal networks – is one approach that can be adopted to better qualify actual – and not only planned or perceived – care coordination.

This is facilitated by the increased availability of health administrative data for research purposes which enables the development of studies at larger scale and lower cost to complement research carried out using survey data [17]. Seminal empirical previous research from Barnett and colleagues in the US has demonstrated that shared patients among healthcare providers in medical claims represent a conduit for the diffusion of information and are a predictor of important working relationships (such as exchange of clinical advice or informal establishment of common referral pathways) [18]. Other applications have emerged, in particular in North America and Australia [19–35], but none has specifically focused on mental healthcare [36]. In France, despite the availability of linked claims data covering hospital and community healthcare, this field of research has been little developed.

In this context, we identify and characterize existing types of healthcare provider patient-sharing networks for severe mental health disorders in one region of France, prior to the PTSM reform, using health administrative data. We then analyse the association between the characteristics of these networks and the quality of the care they provide.

## Methods

### Research framework

Our research is based on quantitative network analysis [37], which provides a mathematical framework to study care coordination at the population level. We consider that health systems can be summarized as bipartite networks, i.e. networks involving two sets of actors: persons needing care and healthcare providers. Each time a person visits a provider, this creates a link between the two sets of actors. This patient flow can then be used to identify groups of healthcare providers with common patients, which ultimately constitute healthcare provider patient-sharing networks [23, 26]. This is this type of one-mode networks which we studied here, and not their bipartite structure. Our research, which focuses on severe mental health disorders, relies on two main hypotheses. The first is that studying the characteristics of these networks provides insight regarding the coordination patterns of healthcare providers within the networks. The second is that specific characteristics of healthcare provider networks (in particular in terms of structure and composition) are related to better quality of care. Both hypotheses are supported by previous research [14, 19, 27].

**Fig. 1:**
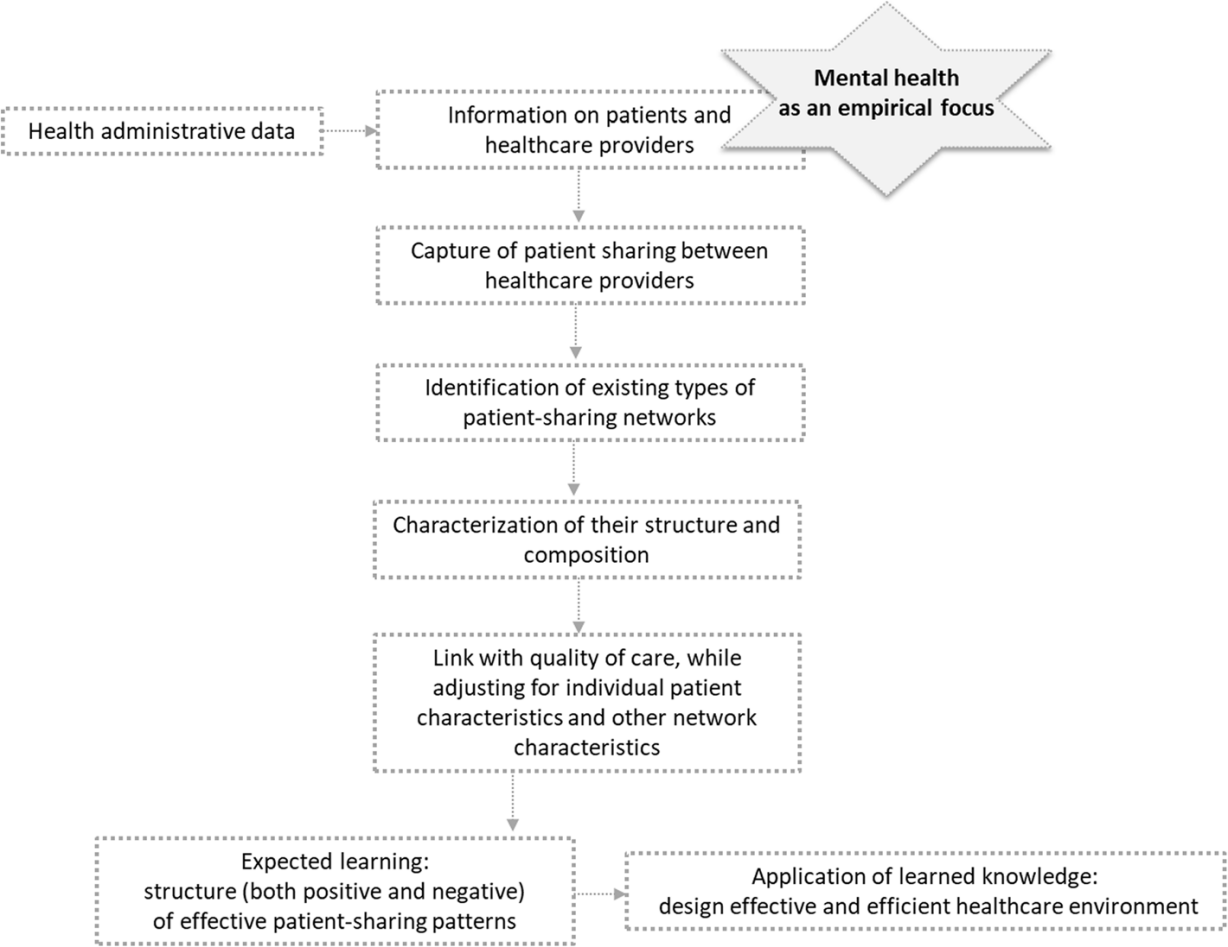
Research framework

The main features of our research framework, adapted from Uddin et al. [32] are presented in Fig. 1.

### Setting

Our study was carried out in an entire region of France, the *Provence-Alpes-Côte-d’Azur* (PACA) region. Close to 5 million inhabitants live in this region which includes Marseille, the second most populous French city. However, this region, which is highly touristic, presents significant contrasts in terms of urbanicity and population density with a strong coastal anchorage, few inhabitants in rural and alpine regions and variations by season. Similarly, healthcare supply is concentrated on the coast while medically underserved areas remain in the hinterland. Overall, the percentage of self-employed community-based health professionals is far higher than the national average: 23% higher for general practitioners (GPs), 51% higher for medical specialists and 185% higher for nurses. Healthcare consumption is also 16% higher than the national average, which could be linked to an aging and deprived population: the PACA region has the fifth highest aging index and is the third poorest region in France with strong social and economic inequalities [38]. In this region, 19 public and private non-profit hospitals participate in the territorial organization of mental healthcare (psychiatric sectorization) for adult patients. Six (32%) are specialized in psychiatry and represent 64% of all inpatient psychiatric beds. An additional 26 private hospitals provide mental healthcare on the territory [39].

### Data sources

Data on patient visits to healthcare providers were extracted from the French national health data system (“*Système national des données de santé*”, SNDS) which contains all billing records from the social health insurance (SHI) which currently covers almost 100% of the resident population [40]. This database provides comprehensive information on healthcare utilization in community-based settings and in public and private hospitals as well as individual information on the socio-demographic and medical characteristics of patients [41]. Other databases were used more sporadically to obtain descriptive aggregated information on healthcare providers. They included the annual national survey on healthcare providers (“*Statistique annuelle des établissements de santé*”, SAE) and the national register of health and social institutions (“*Fichier national des établissements sanitaires et sociaux*”, FINESS) [42, 43].

All these databases are characterized by their exhaustivity and are managed either by the social health insurance fund (SNDS database) or the French ministry of health (SAE and FINESS databases).

### Construction of healthcare provider networks

#### Study patients

Patients included in our study were adults with a diagnosis of severe mental health disorder. They were admitted in full-time inpatient care (used as a further severity criterion) in a psychiatric department of a public or private non-profit hospital of the PACA region during the year 2012 or 2013 (index hospitalization), whatever their zip code of residence. Our definition of severe mental health disorder was based on the international literature [44] and validated with psychiatrists. Disorders included schizophrenia, schizotypal and delusional disorders (F2x of the International classification of diseases, tenth revision (ICD-10) [45]); manic episode, bipolar affective disorder and emotionally unstable personality disorder (F30x, F31x and F603); and severe depressive episode or current severe episode of a recurrent depressive disorder with or without psychotic symptoms (F322, F323, F332, F333). Patients over 65 years old were excluded due to the specific health needs of this population [46]. Patients with an index hospitalization in the two military hospitals of the PACA region were also excluded due to the impossibility of obtaining exhaustive healthcare contacts for this population. We reported patients’ demographics (age and sex) as well as an individual-level indicator of economic deprivation: whether the patient was included in the scheme covering healthcare costs for low-income groups (“*couverture maladie universelle complémentaire”*, CMU-C). Furthermore, we considered clinical characteristics: diagnostic group of the main severe mental health disorder, precedence of this disorder as shown by a contact in hospital-based settings for psychiatric care in the two previous years and presence of a chronic medical comorbidity as shown by inclusion in the somatic categories of the long-term illness scheme for chronic disorders (“*affection de longue durée*”, ALD).

#### Healthcare providers

Once all eligible patients were selected, we focused on their contacts with the healthcare providers who were most likely to be involved in the care of their mental health disorders. This list was defined prior to the start of the study upon discussion with mental care and research specialists, and representatives of the PACA regional health agency. Included contacts were visits to self-employed community-based psychiatrists, GPs and nurses. They further included admissions to hospitals with an activity in psychiatry and admissions to other hospitals for psychiatric conditions, suicide attempts or emergency care not precursor to subsequent hospitalizations. Unlike the index admission, these visits were considered whatever the type of hospitals (public, private non-profit or private for-profit). Obtaining information on salaried health and allied health professionals within hospitals was not possible and we therefore considered hospitals as single provider entities. We also focused on healthcare providers located in the PACA region to avoid spurious contacts for care received when traveling.

#### Process of network identification

Healthcare provider networks for the care and follow-up of severe mental health disorders were identified through contacts between patients and healthcare providers to link pairs of providers who shared patients. We considered one-year time windows following the end of the index hospitalization to allow time for these contacts to develop.

**Fig. 2:**
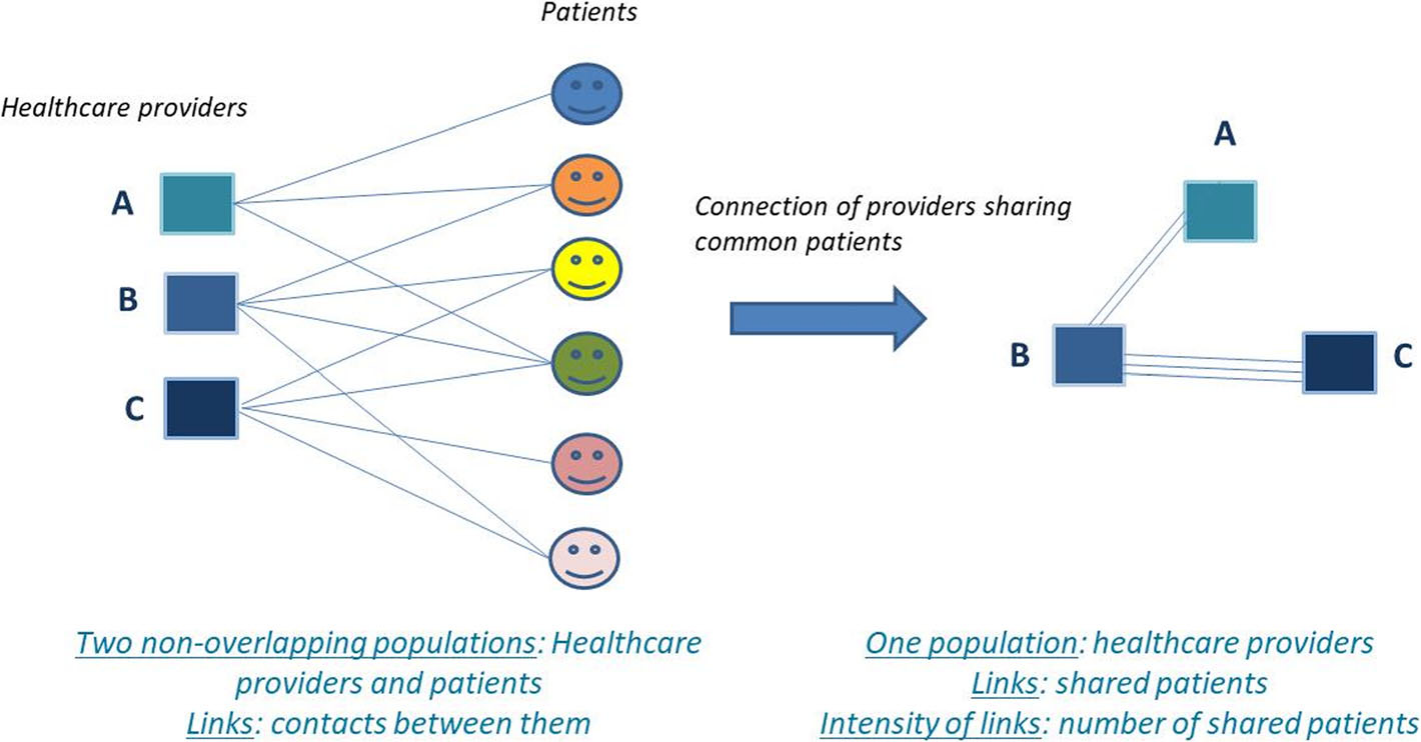
Bases of identification of healthcare provider patient-sharing networks

Links between healthcare providers were weighted by the number of shared patients. The bases of the identification process of healthcare provider patient-sharing networks are summarized in Fig. 2.

As mental healthcare is still very hospital-centered in France [9] and as we only included patients with severe mental health disorders who were hospitalized at least once, we specifically focused on patient sharing between the hospital of patients’ index hospitalization (index hospital) and other providers as well as between these other providers when they were connected to the same index hospital.

We focused on the strongest links by setting a minimal relative threshold of patients shared to be included in the healthcare provider networks. This avoided considering spurious contacts unlikely to correspond to recurrent information-sharing and referrals between the index hospital and other providers, and improved specificity and sensitivity [18]. The threshold was set as being strictly superior to the third quartile of the distribution of the number of shared patients calculated between the index hospital and each type of provider separately. We therefore retained in each network only hospitals that shared more than six patients with the index hospital, GPs that shared at least three patients with the index hospital, and psychiatrists and nurses that shared at least two patients with the index hospital. Within the PACA region, networks were not constrained geographically to avoid artificial boundary specifications which can impede identification of the actual structure of healthcare provider networks [36].

#### Characterization of networks

- Structural characteristics

Structural characteristics of the healthcare provider patient-sharing networks included the density of each network, defined as the proportion of all possible links that are actually present in a network of a given size. We also calculated transitivity, which is a measure characterizing the balance of links within a triad, i.e. the probability that two providers connected to the same provider are also connected [37, 47, 48]. These measures define the strength of cohesion within the networks [48]. They were expressed on a scale ranging from 0 to 100. We also calculated a centrality measure, the weighted degree, which represents the number of adjacent links for each provider taking into account link intensity [37]. This indicator was synthetized at the network level by its median value across all providers of the network. Finally, we included the number of providers in the networks, excluding the index hospital, and the number of links between providers within the networks. These indicators have been used previously in the recent health services research literature [14, 36, 49].

- Compositional characteristics

We calculated indicators characterizing the composition of healthcare provider networks. We included the type of index hospital (specialized or not in psychiatry) and its number of psychiatric inpatient beds. We also focused on differentiation (number of different services offered within a network) [50] by calculating the percentage of the different types of providers (GPs, psychiatrists, nurses, hospitals) within each network, and their number (for community-based health professionals only) per 100,000 adult inhabitants served by each network. This population was defined as the population (aged 20 to 65 years old) of all the catchment areas served by the index hospital in the frame of the territorial organization of mental healthcare in France (psychiatric sectorization) [11].

- Other characteristics

To account for the lack of data on contacts with social care in the SNDS database, we characterized the overall availability of all types of mental care in the vicinity of the index hospital. For this purpose, we used a national typology of care which classifies French local counties (“*départements*”) into five categories based on the availability and diversity of the different types of mental care (including social care) [51]. To adjust for the fact that follow-up contacts provided by hospital teams within the index hospital were not used to build networks, we considered the percentage of specialized ambulatory care provided in the index hospital settings since psychiatric ambulatory care centers managed by hospitals are very common in France. We also added the average number of contacts with any healthcare provider per patient in each network to take into account the intensity of contact (including those within the index hospital) and the fact that some patients may not have any contact at all. Finally, we calculated a loyalty index measuring the share of patients with an index hospitalization in a given hospital who were living in the official catchment area of this hospital.

### Quality of care within the networks

Quality of care in a healthcare provider patient-sharing network was characterized using a set of indicators measured on all the patients with an index hospitalization for schizophrenia, schizotypal or delusional disorder in the index hospital of the network. The restriction of the measure of quality of care to this diagnostic group was motivated by the necessity to define tailored quality indicators based on clinical recommendations for a homogenous population. As healthcare quality is a complex multi-dimensional concept [52], we considered a complementary set of utilization-based quality metrics corresponding to four different domains. They were adapted from previous work on quality of care [20, 53, 54] to specifically relate to the ability of the health system to maintain and improve the health of patients with mental health disorders. The considered domains included: 1. *Frequency of psychiatric inpatient care* through an indicator measuring the presence of at least one readmission within 15 days of the index hospitalization; 2. *Hospital-community transitions* through an indicator measuring the presence of at least one contact with a community-based physician designated by the patient as his/her referring physician (“*médecin traitant*”) in the frame of coordinated healthcare pathways [40] within 2 months of the index hospitalization; 3. *Access to somatic care in the community* through an indicator measuring the presence of three recommended prevention procedures,^1^ as supported by the guidelines of the French psychiatric federation for improving the somatic care of patients with severe and persistent mental illnesses [55], within 2 years of the index hospitalization; 4. *Evidence-based medications* through an indicator measuring the presence of at least nine deliveries of antipsychotic drugs within twelve months of the index hospitalization (based on the minimum number of deliveries necessary to obtain the continuity of drug treatment which is recommended after a hospitalization [56]).

### Statistical analyses

#### Description of the study patients

We carried out a descriptive analysis of the demographic, clinical and socio-economic characteristics of the population used to build healthcare provider patient-sharing networks, including a sub-analysis for patients with schizophrenia or schizotypal or delusional disorders for whom quality indicators were specifically measured.

#### Identification of the main types of healthcare provider patient-sharing networks

Hierarchical clustering, using principal component analysis (PCA) [57], was conducted to create a typology of the identified networks. This has been frequently used as a way to identify and synthetize core characteristics of healthcare provider networks in the literature [50, 58]. In the PCA, quantitative characteristics of networks were used as active variables and their qualitative characteristics as illustrative variables. The final types of healthcare provider patient-sharing networks selected were those that were robust to the choice of clustering method (Ward’s clustering, average-link clustering and k-means). We then described variations in the characteristics of networks across the different clusters identified. We focused in particular on the quantitative variables that were the most significantly associated with each cluster. We also took into account variations in the size of networks which can strongly influence their structural characteristics. We also visualized the parangon (healthcare provider network with the coordinates closer to the center of the cluster) using the Fruchterman Reingold layout algorithm [59].

#### Characteristics of healthcare provider patient-sharing networks associated with quality of care

To identify the characteristics of healthcare provider patient-sharing networks associated with quality of care indicators, we first described variations in these indicators when aggregated across the previously identified clusters of networks. We then conducted multivariable analysis. Four separate models were run using each of the four quality indicators included as the dependent variable. They were introduced at the individual patient level to avoid the loss of information associated with data aggregation [60]. The explanatory variables in the model, in line with the objectives of our research, were the characteristics of healthcare provider patient-sharing networks, linked in particular to their structure and composition, after testing for correlation among these variables. We also introduced patient-level variables (demographics, economic deprivation and clinical characteristics) as adjustors as these are often associated with quality of care. All independent variables were binary and common in our study population. We therefore carried out log-binomial models to express the effects in terms of relative rates (RR) with 95% confidence intervals (95% CI) [61]. As correlation can be expected in the data of patients seen in the same cluster of healthcare provider networks, generalized estimating equations (GEE) models were used to relax the assumption of independence at the cluster level [19, 26, 29, 62]. We chose not to carry out multi-level models with cluster as a random effect since previous research has questioned their reliability for a number of clusters inferior to 20, with the risk of obtaining a null variance [63]. This was indeed observed when attempting preliminary exploratory empty multi-level models on our four quality indicators. Finally, to assess the impact of the use of a minimal relative threshold of shared patients to include a given provider in the different networks, we carried out a sensitivity analysis by replicating the multivariable analysis without applying any threshold.

### Software

SAS 9.4. (SAS Institute Inc., Cary, NC, USA) was used for data cleaning and management as well as for multivariable analyses (using the GENMOD procedure with a log link, binomial distribution and repeated statement for clusters of networks). Construction and characterization of healthcare provider patient-sharing networks and clusters were performed in R version 3.4.4, using the RStudio interface, and the FactoMineR (version 1.40), NbClust (version 3.0) and igraph (version 1.1.2.) packages. A statistical significance level of 5.0% was used throughout the analysis.

## Results

### Study patients

9454 patients matched our inclusion and exclusion criteria. 57% were female and 95% lived in the PACA region. A majority (66%) had a diagnosis of schizophrenia or schizotypal or delusional disorder. This specific subgroup was similar to the entire study population (Table 1).

**Table 1:** Study patient characteristics

	Full patient population (*n* = 9454)	Patients with a diagnosis of schizophrenia or schizotypal or delusional disorder (*n* = 6279)
**Characteristic**	**Mean (SD) or n (%)**	**Mean (SD) or n (%)**
*Demographic characteristics*		
Age	41.7 (12.2)	40.2 (11.9)
Sex (male)	5395 (57.1)	4145 (66.0)
*Clinical characteristics*		
With a diagnosis of schizophrenia or schizotypal or delusional disorder	6279 (66.4)	–
With a diagnosis of severe depressive episode or current severe episode of a recurrent depressive disorder	1084 (11.5)	–
With a diagnosis of manic episode, bipolar affective disorder or emotionally unstable personality disorder	2091 (22.1)	–
Precedence of the main psychiatric disorder	5311 (56.2)	3792 (60.4)
Inclusion in the long-term illness scheme for somatic disorders	1432 (15.2)	842 (13.4)
*Socio-economic characteristics*		
Inclusion in the scheme covering healthcare costs for low-income groups	1950 (20.6)	1285 (20.5)

### Construction of healthcare provider patient-sharing networks

Study patients had an index hospitalization in the 19 hospitals of the PACA region participating in the territorial organization of mental healthcare (psychiatric sectorization). 19 healthcare provider patient-sharing networks, centered on the hospitals of the index hospitalizations, were built. In total, 67 hospitals (in addition to the index hospitals), 225 psychiatrists, 856 general practitioners and 532 nurses were included in the networks based on the distribution of links by type of provider (versus respectively 75; 587; 4283; and 3607 without applying any relative threshold).

### Main types of healthcare provider patient-sharing networks

The hierarchical cluster analysis identified three main types of healthcare provider patient-sharing networks. The first cluster (C1) included networks (*n* = 5) which were characterized by a significantly higher average percentage of specialized ambulatory care provided in the index hospital than in other clusters. They shared patients with few private specialized healthcare providers: the proportion of private hospitals among hospital providers was significantly lower than in other clusters and few psychiatrists were involved among community-based providers. The loyalty index was consistently high in all the networks of C1. Most of them were located either in rural territories or in areas with limited healthcare resources. These networks were also characterized by a high average density and transitivity which was not only linked to low network sizes as the average total number of providers in the networks of the first cluster, while limited, was higher than that of the third cluster.

The second cluster (C2) included networks (*n* = 9) which, in opposition with C1, presented a significantly lower average percentage of specialized ambulatory care provided in the index hospital. They also presented a significantly higher median average degree and higher average number of contacts with any healthcare provider per patient. They were all located in dense urban areas with strong healthcare supply. Some networks of this cluster, whose index hospitals were located in the same most populated cities, presented low values for the loyalty index. These networks were also characterized by a low average density and transitivity but a high average total number of providers per network, with the involvement in particular of community-based psychiatrists and nurses.

The third cluster (C3) included networks (*n* = 5) where the average number of contacts with any healthcare provider per patient was significantly lower than in other clusters and patient-sharing was mostly directed towards physicians in the community who represented a significantly higher share of providers involved than in other networks. Similarly to networks of C2, they were all located in areas with strong healthcare supply but the index hospitals were located in mid-sized cities and presented higher loyalty index. Average density and transitivity were rather low in comparison to other clusters when taking into account the small average total number of providers within networks.

**Fig. 3:**
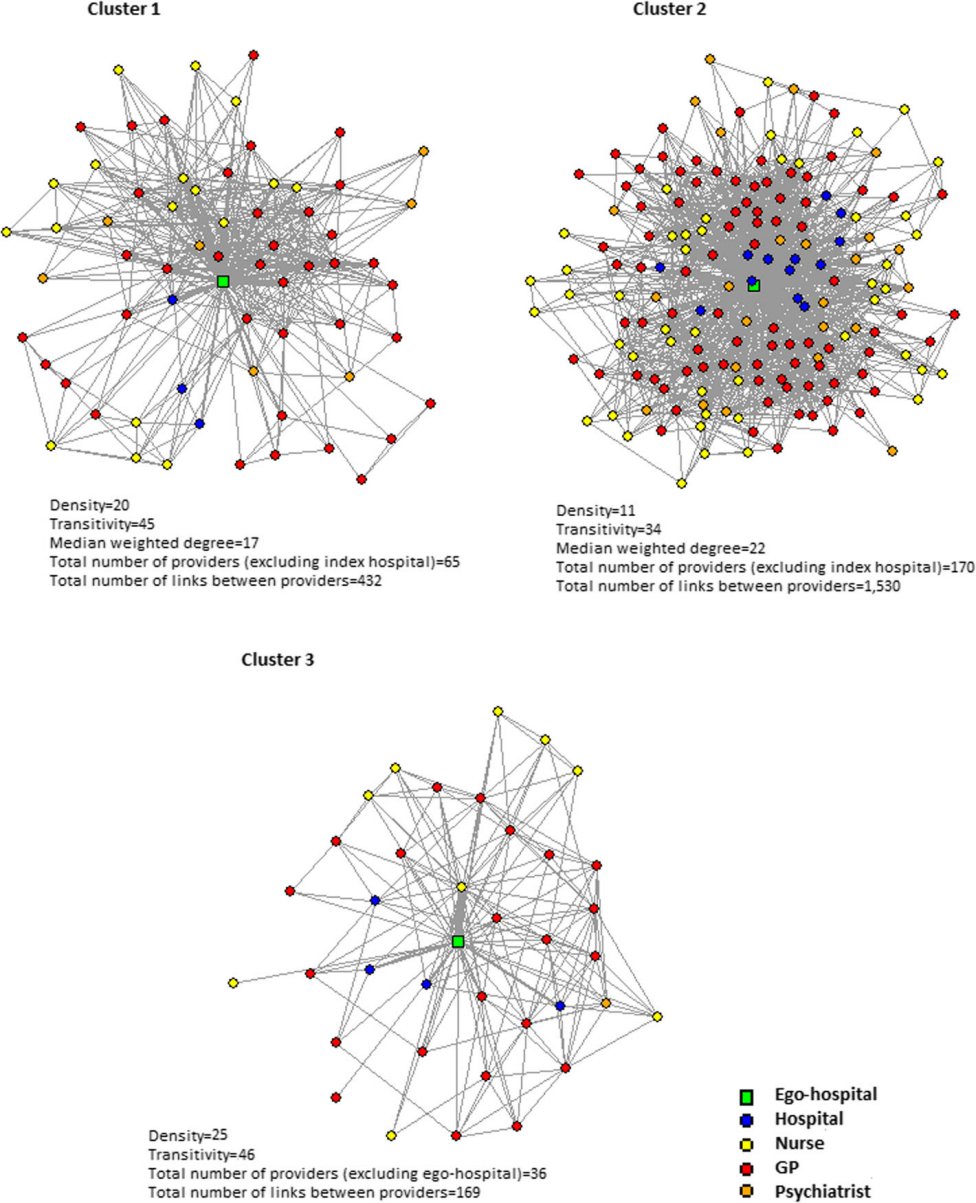
Vizualization of the parangon network of each cluster. Note: In these illustrations, healthcare providers are depicted as nodes and shared patients between them as links. The thickness of the links is proportional to the number of shared patients. Networks were drawn with the R igraph package version 1.1.2. using the Fruchterman Reingold layout algorithm which distributes nodes evenly making lengths of links uniform and reflecting symmetry [59]

Detailed characteristics of each cluster are presented in Table 2 and the healthcare provider patient-sharing networks identified as the parangon of each cluster are illustrated in Fig. 3.

**Table 2:** Characteristics of identified types of healthcare provider patient-sharing networks

Characteristic		Mean (SD) [or n (%) when specified]		
		C1 (*n* = 5)	C2 (*n* = 9)	C3 (*n* = 5)
*Structural characteristics*				
Density^a^		24.09 (15.45)	14.27 (6.28)	19.49 (7.25)
Transitivity		44.94 (16.61)	32.51 (7.83)	37.17 (10.36)
Median weighted degree^a^		16.60 (4.83)	19.89 (6.01) ^c^	13.20 (2.36)
Total number of providers (excluding index hospital)		85.00 (91.67)	124.56 (82.75)	59.60 (52.77)
Number of links between providers		534.80 (619.17)	994.67 (809.32)	315.20 (374.18)
*Compositional characteristics*				
Index hospital specialized in psychiatry^b^, n (%)		1.00 (20.00)	4.00 (44.44)	1.00 (20.00)
Number of inpatient psychiatric beds in the index hospital		150.60 (167.49)	159.00 (109.32)	104.40 (127.52)
Number per 100,000 adult inhabitants of community-based providers	Psychiatrists^a^	7.07 (4.42)	10.19 (6.77)	5.19 (2.80)
	GPs^a^	40.18 (17.40)	32.05 (13.04)	23.81 (5.50)
	Nurses	24.04 (6.72)	21.66 (6.90)	9.94 (3.35)
% of the different types of providers	Psychiatrists	8.72 (4.54)	13.99 (6.98)	11.77 (6.16)
	GPs	50.61 (8.65)	44.51 (4.85)	53.79 (10.14)
	Nurses	31.33 (6.86)	31.49 (5.42)	22.62 (7.45)
	Hospitals^a^	9.34 (4.81)	10.01 (3.03)	11.82 (2.02)
% of specialized health professionals (psychiatrists) among community-based health professionals		9.63 (5.21)	15.54 (7.80)	13.37 (7.00)
% of physicians among community-based health professionals^a^		65.48 (7.01)	64.95 (6.35)	74.44 (8.27) ^c^
% of private hospitals among hospital providers^a^		6.86 (9.60) ^c^	47.21 (9.39)	52.78 (17.79)
*Other characteristics*				
Typology of mental care in the vicinity of the index hospital^b^	Areas with limited healthcare resources, n (%)	1.00 (20.00)	0.00 (0.00)	0.00 (0.00)
	Areas with resources concentrated in hospital settings, n (%)	0.00 (0.00)	0.00 (0.00)	0.00 (0.00)
	Areas with strong private care supply and an organization adapted to urban practices, n (%)	0.00 (0.00)	5.00 (55.56)	4.00 (80.00)
	Areas with strong healthcare resources, n (%)	2.00 (40.00)	4.00 (44.44)	1.00 (20.00)
	Mostly rural areas combining healthcare and health and social care supply n (%)	2.00 (40.00)	0.00 (0.00)	0.00 (0.00)
Loyalty index^b^	> 80%, n (%)	5.00 (100.00)	6.00 (66.67)	3.00 (60.00)
	[60–80%], n (%)	0.00 (0.00)	1.00 (11.11)	2.00 (40.00)
	< 60%, n (%)	0.00 (0.00)	2.00 (22.22)	0.00 (0.00)
% of specialized ambulatory care provided in the index hospital^a^		36.10 (14.27) ^c^	16.90 (8.22) ^c^	27.70 (6.84)
Average number of contacts with any healthcare provider per patient^a^		65.41 (5.56)	81.72 (12.40) ^c^	47.74 (4.08) ^c^

^a^Variables used as active variables in the PCA. Density, transitivity, number of providers, number of links between providers and number of inpatient psychiatric beds in the index hospital were highly correlated; thus only density was introduced in the PCA. The proportion of nurses and the proportion of GPs among the healthcare providers of the network were highly correlated with the percentage of physicians among community-based health professionals in the network, and were therefore not introduced in the PCA. The proportion of psychiatrists among the healthcare providers of the network and the proportion of specialized health professionals among community-based health professionals were highly correlated with the number of psychiatrists in the network per 100,000 inhabitants, and were therefore not introduced in the PCA

^b^Variables used as illustrative variables in the PCA

^c^Significantly different from the overall mean

### Quality of care and association with network characteristics

Few variations in quality of care indicators were observed when aggregated at the cluster level. Indicators of frequency of inpatient care and hospital-community transitions were the highest in cluster 1, while indicators of access to somatic care in the community and of evidence-based medications were the lowest in this cluster. The latter was the only indicator for which the difference observed with the overall mean across clusters was statistically significant (Table 3).

**Table 3:** Quality indicators across network clusters

Indicator^a^	Mean (SD)		
	C1 (*n* = 5)	C2 (*n* = 9)	C3 (*n* = 5)
% of patients with 15-day readmissions in psychiatric inpatient care	32.14 (8.39)	28.99 (7.13)	28.17 (3.81)
% of patients with at least one contact with their referring physician within two months of the index hospitalization	33.34 (6.89)	32.55 (4.91)	32.66 (4.50)
% of patients with three recommended somatic prevention procedures within two years of the index hospitalization	45.83 (10.00)	53.15 (5.44)	49.30 (3.82)
% of patients with at least nine deliveries of antipsychotic drugs within one year of the index hospitalization	47.30 (9.32) ^b^	58.00 (4.43)	56.21 (4.73)

^a^Measured on patients with an index hospitalization for schizophrenia or schizotypal or delusional disorder

^b^Significantly different from the overall mean

In the multivariable analysis, several associations between quality of care indicators and a number of network structural and compositional characteristics presented *p*-values inferior to 0.05. However, effect sizes were consistently weak with relative rates close to 1, except for the specialization in psychiatry of the index hospital which was negatively associated with 15-day readmissions in psychiatric inpatient care (RR: 0.58, 95% CI: 0.52–0.66).

Detailed results of the multivariable analysis, including associations found with adjustors, are presented in Table 4.

**Table 4:** Results of the multivariable analysis for each quality indicator

Quality indicator Characteristic^a^		Readmission	Contact with the referring physician	Three recommended prevention procedures	At least 9 deliveries of antipsychotic drugs
		RR (95% CI)	RR (95% CI)	RR (95% CI)	RR (95% CI)
*Patient characteristics*					
Age		1.00 (0.99–1.00) ^b^	1.01 (1.01–1.02) ^b^	1.02 (1.01–1.02) ^b^	1.00 (1.00–1.00) ^b^
Sex (male vs. female)		1.12 (1.10–1.14) ^b^	0.79 (0.75–0.83) ^b^	0.82 (0.79–0.85) ^b^	0.97 (0.95–1.00) ^b^
Inclusion in the scheme for low-income groups (vs. non-inclusion)		1.10 (1.03–1.17) ^b^	0.87 (0.83–0.92) ^b^	1.00 (0.97–1.03)	0.84 (0.79–0.89) ^b^
Precedence of the mental disorder (vs. no precedence)		1.23 (1.13–1.33) ^b^	0.83 (0.75–0.90) ^b^	0.97 (0.93–1.01)	1.08 (1.06–1.10) ^b^
Inclusion in the long-term illness scheme for somatic disorders (vs. non-inclusion)		1. 01 (0.94–1.08)	1.39 (1.27–1.51) ^b^	1.26 (1.15–1.37) ^b^	1.03 (0.99–1.06)
*Network structural characteristics*					
Density		0.96 (0.95–0.96) ^b^	1.02 (1.00–1.03)	1.03 (1.01–1.04) ^b^	1.02 (1.00–1.03) ^b^
Median weighted degree		0.99 (0.97–1.01)	1.00 (0.97–1.04)	1.03 (1.01–1.05) ^b^	1.03 (1.01–1.06) ^b^
*Network compositional characteristics*					
Type of index hospital (specialization in psychiatry vs. general hospital)		0.58 (0.52–0.66) ^b^	0.98 (0.90–1.07)	1.01 (0.96–1.06)	0.95 (0.89–1.00)
% of physicians among community-based health professionals		0.96 (0.95–0.97) ^b^	1.03 (1.01–1.05) ^b^	1.01 (1.00–1.03) ^b^	1.01 (1.00–1.02)
% of hospitals among all healthcare providers		1.05 (1.03–1.07) ^b^	0.92 (0.90–0.94) ^b^	0.98 (0.96–1.00) ^b^	0.99 (0.98–1.00)
% of private hospitals among hospital providers		1.00 (1.00–1.00)^b^	1.00 (1.00–1.00)	1.00 (1.00–1.00) ^b^	1.00 (0.99–1.00) ^b^
Number of psychiatrists in the network per 100,000 inhabitants		0.99 (0.99–1.00) ^b^	0.99 (0.98–0.99) ^b^	1.01 (1.01–1.01) ^b^	0.99 (0.99–1.00) ^b^
Number of GPs in the network per 100,000 inhabitants		1.01 (1.00–1.02)	0.98 (0.97–0.99) ^b^	0.98 (0.97–0.99) ^b^	0.99 (0.98–1.00) ^b^
*Other characteristics at the network level*					
Typology of mental care in the vicinity of the index hospital (reference: mostly rural areas which combine healthcare and health and social care)	Areas with limited healthcare resources	0.12 (0.08–0.19) ^b^	1.24 (0.51–3.01)	2.95 (1.62–5.36) ^b^	2.87 (1.41–5.82) ^b^
	Areas with strong private care supply and an organization adapted to urban practices	0.27 (0.19–0.39) ^b^	0.91 (0.44–1.89)	3.14 (2.05–4.79) ^b^	2.80 (1.64–4.78) ^b^
	Areas with strong healthcare resources	0.25 (0.17–0.38) ^b^	1.18 (0.54–2.61)	3.81 (2.23–6.50) ^b^	3.09 (1.68–5.68) ^b^
Loyalty index (reference: < 60%)	> 80%	0.43 (0.31–0.61) ^b^	1.32 (0.64–2.71)	2.17 (1.43–3.28) ^b^	2.55 (1.62–4.02) ^b^
	[60–80%]	0.50 (0.36–0.69) ^b^	1.36 (0.69–2.70)	1.87 (1.27–2.76) ^b^	2.40 (1.57–3.67) ^b^
% of specialized ambulatory care provided in the index hospital		0.99 (0.99–0.99) ^b^	0.99 (0.98–1.00) ^b^	1.00 (1.00–1.00) ^b^	1.00 (1.00–1.01)
Average number of contacts with any healthcare provider per patient in the network		0.98 (0.98–0.99) ^b^	1.01 (1.00–1.01) ^b^	1.01 (1.00–1.01) ^b^	1.01 (1.00–1.01) ^b^

Note: *RR* relative risk, *CI* confidence interval

^a^See legend of Table 2 for correlations which explained that some of our explanatory variables of interest were not introduced in the models

^b^Statistically significant

After replicating the multivariable analysis without applying any minimal threshold of shared patients to include providers in the different networks, very little changes were observed in the relative rates for network structural and compositional characteristics. They remained close to 1 – except for the type of index hospital in the model of readmissions in psychiatric inpatient care (RR: 0.68, 95% CI: 0.60–0.99) – as was observed in the main multivariable analysis.

## Discussion

Our research provides a first look at the structure and composition of healthcare provider patient-sharing networks for the care of severe mental health disorders in one region of France. Public and private non-profit hospitals with an activity in psychiatry appeared to develop different types of patient-sharing patterns with other providers. The first type corresponded to healthcare provider networks strongly organized around the main hospital providing psychiatric care, acting as the unique mental healthcare provider in the vicinity, according to the logic of psychiatric sectorization. The second type corresponded to scattered networks involving numerous and diverse healthcare providers in dense urban areas, and the third type corresponded to medically-oriented healthcare provider networks involving mainly physician providers. The delivery of antipsychotic drugs appeared to be less concordant with evidence-based guidelines at the aggregated level in the first type of healthcare provider patient-sharing networks. However, we did not find any strong association between structural or compositional characteristics of networks and quality indicators in the multivariable analysis with the exception of the specialization or not in psychiatry of the index hospital.

Previous research, using the same methodological approach, consistently found variations in the patient-sharing patterns of healthcare providers [48, 64]. Prior studies also demonstrated that meaningful similarities existed across many evolving organizations within health systems despite the perception that organizational changes in healthcare are chaotic [50]. The types of healthcare provider patient-sharing networks identified in our study could serve as a basis for the formal constitution of coordination networks, notably in the frame of the PTSM territorial networks for mental care made compulsory by recent national policies [12]. Explicit structuring of care delivery processes building on pre-existing informal working relationships between care providers is likely to be easier to develop. It can also be hypothesized to enable efficiency and acceptability among providers and to retain the majority of care within the networks [26]. In the mental health field, pre-existing characteristics of healthcare provider networks were also found to impact the implementation of a reform supporting strong coordination across all types of mental care in Canada [16]. This suggests that in some types of healthcare provider networks identified in our study the implementation of the PTSM reform might be more challenging and require specific attention. This is in particular the case of healthcare provider networks for which preferential patient-sharing patterns were either very centred on public psychiatry or on physician providers. Implementation strategies should be adapted to the extent of the hegemonic position of the index hospital on the territories on which these healthcare provider networks formed and to the characteristics of these territories (especially in terms of urbanicity and availability of all types of mental care supply) which were homogenous within each different type of networks.

Regarding the link between the characteristics of healthcare provider patient-sharing network and the care provided within these networks, there is emerging evidence that patients seen in broader and more disconnected networks receive lower quality care and have higher costs for the treatment of specific somatic disorders [65, 66]. In our study, we found few significant associations between the structure and composition of healthcare provider networks and indicators of quality of care which suggest that providers’ patient-sharing patterns might not be the main driver of optimal care provision in the context explored. Recent research carried out in the mental health field has underscored the challenge of identifying the optimal structure of such networks which varied with the outcomes considered. In particular, network characteristics required to achieve optimal care provision differed from those required to enable an optimal social integration of patients [14]. While we specifically focused on indicators of quality of care in our study, the only characteristic of networks with a significant impact (specialization or not in psychiatry of the index hospital) was not consistently associated with all indicators considered. This suggests that formal healthcare provider network structure should be adapted to the key policy priority they are trying to achieve. This might be a challenge in the frame of the PTSM reform which has many goals, ranging from early detection of mental health disorders to patients’ personal and social recovery [12].

Our findings should be interpreted in light of two main types of limitations. First, there are limits linked to the study design since we examined healthcare provider patient-sharing networks using only a snapshot in one region of France which might not be fully generalizable to all French practice. Furthermore, if the methods we present here can be reproduced and adapted to the specificities of different contexts, our findings might not be fully transferable to other disorders or countries where care has not so strongly put hospitals at its core. Previous research has also found that the involvement of primary or specialized care providers for mental health disorders was highly dependent on organizational factors at the health system level, which should be taken into account before any extrapolation [67]. In addition, our findings should be carefully interpreted in the context of our analytic approach. In particular, a host of factors can influence patient sharing between providers. These include for instance access to care, geographical proximity, relatives’ preferences or health literacy [35]. However, our hypotheses were not based on what might have motivated this patient sharing and we were not focusing on the description of underlying mechanisms that could explain network formation. Even when a shared patient is not the result of a referral, providers who share patients may learn what others are doing for the patients, which may in turn influence their practice style. Second, there are limitations linked to the data used for our research. In particular the construction of quality metrics using health administrative data is prone to measurement error and does not enable to assess all key indicators such as the patient’s personal and social recovery. It is also possible that there is unobserved confounding by unmeasured disease severity and comorbidities. Regarding the identification of care providers, no information was available on social care or private psychologists as they are not reimbursed by the SHI [68]. While we adjusted for a national typology of all types of mental care on French territories [51], it might have been more accurate if we were able to integrate these actors in our networks. Similarly, we had no information on individual health professionals within hospitals, which can explain the relatively high density observed in our study in comparison to others [23]. However, such professionals can be hypothesized to have similar patient-sharing patterns based on their common working place, and this limitation was true for all hospitals.

Despite these limitations, our study has several strengths. It explores the applicability and specificities of an emerging and novel methodological approach, based on health administrative data, outside the North American context and for a new type of population, that of individuals with severe mental health disorders for which coordination is of key significance. In addition, as the SHI covers the entire French resident population, the health administrative data we used to identify patients and care providers is exhaustive. This is likely to increase the representativeness of our findings.

Several directions for future research can be derived from our work. It would notably be worth studying the stability of identified healthcare provider patient-sharing networks over time and their longitudinal dynamics. In particular, the impact of the creation of the formal PTSM territorial networks for mental care on relationships between care providers should be assessed in a few years. Detecting groups of most strongly connected providers within the existing networks using community identification algorithms is also an important research perspective as they could provide a more precise unit of analysis to assess associations between structural and compositional characteristics of healthcare provider networks and patients’ outcomes. Furthermore, qualitative research might help determine levers linked to patient-sharing patterns to improve the quality of the care provided. The association between network characteristics and quality of care also deserves to be further explored by linking health administrative data with quality indicators which are not limited to process measures but also include patients’ experiences, social and personal recovery, rates of employment or stable housing. Similarly, our research could usefully be complemented by an analysis of the impact of the characteristics of healthcare provider patient-sharing networks on healthcare costs.

## Conclusions

Our research highlights current patient-sharing patterns for mental healthcare using health administrative data in one region of France. Findings provide a basis to develop explicit structuring of mental care delivery based on pre-existing informal working relationships but suggest that healthcare providers’ patient-sharing patterns might not be the main driver of optimal care provision in the context explored. The shift towards a stronger integration of health and social care in the mental health field might impact these results but is currently not observable in the administrative data available for research purpose which should evolve to include social care.

## Data Availability

The datasets generated and/or analyzed during the current study are available from the corresponding author on request and in compliance with requirements from the French data protection authority.

## Abbreviations

ALD: *Affection de longue durée*, Long-term illness scheme for chronic disorders
C1: Cluster 1
C2: Cluster 2
C3: Cluster 3
95% CI: 95% Confidence interval
CMU-C: *Couverture maladie universelle complémentaire*, Scheme covering healthcare costs for low-income groups
FINESS: *Fichier national des établissements sanitaires et sociaux,* National register of health and social institutions
GEE: Generalized estimating equations
GP: General practitioner
ICD-10: International classification of diseases, tenth revision
PACA: *Provence-Alpes-Côte-d’Azur* region
PCA: Principal component analysis
PTSM: *Projets territoriaux de santé mentale,* Formal territorial networks for mental care
RR: Relative rate
SAE: *Statistique annuelle des établissements de santé*, Annual national survey on healthcare providers
SHI: Social health insurance
SD: Standard deviation
SNDS: *Système national des données de santé,* French national health data system

## Footnotes

1 These prevention procedures included electrocardiogram and assessment of renal function combined to at least one other exam among testing of C-reactive protein, thyroid-stimulating hormone, plasmatic lithium, calcemia or beta-hCG for women of childbearing age, exploration of haemostasis or full electrolytogram.
